# DC260126: A Small-Molecule Antagonist of GPR40 that Protects against Pancreatic β-Cells Dysfunction in *db/db* Mice

**DOI:** 10.1371/journal.pone.0066744

**Published:** 2013-06-11

**Authors:** Peng Sun, Ting Wang, Yuren Zhou, Hong Liu, Hualiang Jiang, Weiliang Zhu, Heyao Wang

**Affiliations:** Shanghai Institute of Materia Medica, Chinese Academy of Sciences, Shanghai, China; University of Ulster, United Kingdom

## Abstract

G protein-coupled receptor 40 (GPR40) mediates both acute and chronic effects of free fatty acids (FFAs) on insulin secretion. However, it remains controversial whether inhibition of GPR40 would be beneficial in prevention of type 2 diabetes. This study is designed to evaluate the potential effects of DC260126, a small molecule antagonist of GPR40, on β-cell function following administration of 10 mg/kg dose of DC260126 to obese diabetic *db/db* mice. Oral glucose tolerance test, glucose stimulated insulin secretion and insulin tolerance test were used to investigate the pharmacological effects of DC260126 on *db/db* mice after 21-days treatment. Immunohistochemistry and serum biochemical analysis were also performed in this study. Although no significant change of blood glucose levels was found in DC260126-treated mice, DC260126 significantly inhibited glucose stimulated insulin secretion, reduced blood insulin level and improved insulin sensitivity after 3 weeks administration in *db/db* mice. Moreover, DC260126 reduced the proinsulin/insulin ratio and the apoptotic rate of pancreatic β-cells remarkably in DC260126-treated *db/db* mice compared to vehicle-treated mice (*p*<0.05, n = 8). The results suggest that although DC260126 could not provide benefit for improving hyperglycemia, it could protect against pancreatic β-cells dysfunction through reducing overload of β-cells, and it increases insulin sensitivity possibly via alleviation of hyperinsulinemia in *db/db* mice.

## Introduction

Elevated circulating free fatty acids (FFAs) levels are commonly found in type 2 diabetes and are considered to be one of the most important risk factors for type 2 diabetes [1], [2], [3]. The effects of FFAs on pancreatic β-cells are complex and divergent [3]. Acute administration of FFAs can amplify glucose-stimulated insulin secretion (GSIS) of β-cells under normal circumstances, while prolonged exposure to the saturated fatty acid impairs β-cells function, which is known as lipotoxicity [4].

The G-protein coupled receptor 40 (GPR40) has been found highly expressed in pancreatic β-cells in both human and rodents, which could be activated by medium- and long-chain FFAs [5], [6], [7]. GPR40 mediates both acute and chronic effects of FFAs on insulin secretion and plays an important role in glucose homeostasis [8]. FFAs could activate intracellular Ca^2+^ release via GPR40, but the breakdown of Ca^2+^ homeostasis by sustained elevation of FFA levels could trigger β-cell dysfunction and apoptosis, which might be the mainly cause of FFA-induced lipotoxicity [5], [9]. Steneberg et al. firstly reported that GPR40 knockout mice were resistant to many effects of high-fatty-diet (HFD), such as hyperglycemia, hyperinsulinemia and glucose intolerance. Besides, the diabetic phenotype was observed in transgenic mice overexpressing GPR40 in islets [8]. These results suggest that inhibition of GPR40 may be useful for the prevention and treatment of obesity-associated type 2 diabetes. However, Lan et al. reported that the deletion of GPR40 could not protect mice from HFD-induced metabolic disease [10]. Nagasumi et al. showed that overexpression of GPR40 in β-cells increased insulin secretion and improved glucose tolerance in HFD-treated mice [11]. Moreover, there is clear evidence that GPR40 agonist may be useful in the therapeutic management of hyperglycemia [12], [13]. These inconsistent results indicate that the role of GPR40 in obesity-associated type 2 diabetes still needs further studies.

In previous studies conducted with DC260126 (a novel class of GPR40 antagonist), we have demonstrated that DC260126 could protect MIN6 β-cells from palmitate-induced endoplasmic reticulum (ER) stress and apoptosis [14], [15]. And it could also reduce hyperinsulinemia and improve insulin sensitivity in Zucker fatty rats [16]. In this study, we investigated whether DC260126 is able to prevent β-cells dysfunction in obese diabetic *db/db* mice. It is found that hyperinsulinemia, proinsulin/insulin ratio and the number of apoptotic β-cells were all reduced, and the insulin resistance was improved following 21-days administration of DC260126 in *db/db* mice.

## Materials and Methods

### Animals and treatments

Male C57BL/KsJ-Lep^db^ (*db/db*) and their lean littermates were obtained from Jackson Laboratories (Bar Harbor, Maine, USA). Animals were maintained in a 12 h light-dark cycle at a temperature of 23°C with free access to water and regular chow diet. To investigate the dose-dependent effect of DC260126, nine-week-old *db/db* male mice were divided into four groups (n = 6/group). Mice were give vehicle (5% DMSO in PBS) or DC260126 (3, 10, 30 mg/kg) once daily by tail vein injection for 5 days. At day 5, each group of mice were fasted for 6 h and blood samples were collected from orbital venous plexus and centrifuged for serum separation. Then the concentration of serum insulin level was measured by ELISA kit (Millipore) following its protocol. For long term experiments, six-week-old obese *db/db* male mice were divided into two groups (n = 8/group) and given vehicle (5% DMSO in PBS) or DC260126 (10 mg/kg) once daily by tail vein injection for 24 days, respectively. Meanwhile, their lean littermates were treated with vehicle in an identical manner as normal control. Body weight and food intake were recorded regularly. After 6 h fasting, blood glucose concentrations were monitored by tail vein blood using a glucometer (One-touch Ultra, Lifescan) every week. At the end of the experiment, mice were fasted for 12 h to perform oral glucose tolerance test (OGTT, day 21) and for 6 h to generate insulin tolerance test (ITT, day 23) as described [16] with slight modification indicated in the figure legend. Meanwhile, the insulin release during OGTT was also measured, blood sample was obtained from tail veins and serum insulin concentration was determined by ELISA kit (Alpco, USA). At the end of experiment (day 24), mice were fasted for 6 h, and blood samples were collected from orbital venous plexus and centrifuged for serum separation. Then the animals were killed by CO_2_ inhalation, the pancreas tissue were removed and kept in 4% paraformaldehyde. All of the animal experiments were approved by the Institutional Animal Care and Use Committee in Shanghai Institute of Materia Medica (No: SIMM-2011-01-WHY-04).

### Serum biochemical analysis and proinsulin/insulin ratio measurement

At the end of experiment, serum level of triglyceride (TG) and total cholesterol (TC) was measured by kits (Rongsheng Biotech. Shanghai, China) following their protocols. Serum concentration of proinsulin and insulin were measured by ELISA kit (Alpco, USA) following the protocol provided in the kit, respectively. Proinsulin/insulin (P/I) ratio was calculated. The homeostatic model assessment (HOMA) was calculated as Matthews et al. described [17].

### Immunohistochemistry and TUNEL staining

The entire pancreas from lean and DC260126 or vehicle treated *db/db* mice were removed for paraffin sections, and immunohistochenmistry of insulin and proinsulin were carried out following described methods [18]. At least 5 to 6 sections from each independent individual were analyzed to calculate β-cell mass according to the procedure described [19]. Generally, the mean percentage area of insulin-positive β-cells was determined in each group, and the value was then adjusted for total pancreatic wet weight per animal. Terminal deoxynucleotidyl transferase dUTP nick end labeling (TUNEL) assay, a common method for the determination of cell apoptosis during early stage as it could recognize DNA fragmentation [20], was carried out to evaluate apoptotic pancreatic β-cells rate. Apoptotic cells in pancreas tissue were detected by TUNEL kit (Merck) following its instruction. The rate of apoptosis was calculated as number of TUNEL-positive cells divided with the area of each islet.

### Statistical Analysis

Data were expressed as the mean ± SD. Two-tailed Student’s *t* tests and ONE-WAY ANOVA analysis were performed to determine statistically significant differences. A value of *p*<0.05 was considered significant at the 95% confidence level.

## Results

### Effects of DC260126 on fasting serum insulin (FSI), fasting plasma glucose (FPG) and other pharmacodynamic biomarkers in *db/db* mice

**Figure 1:**
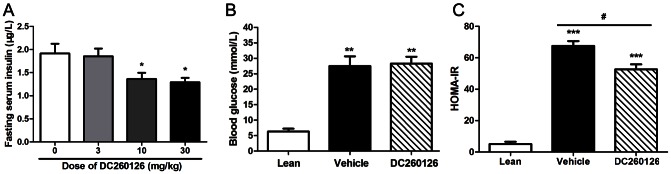
The effect of DC260126 on the FSI, FPG and HOMA-IR in *db/db* mice. (A) DC260126 acute inhibited the FSI level of *db/db* mice dose-dependently after 5 days treatment (n = 6, * *p*<0.05 compared to vehicle-treated group). (B) FPG level was measured after 6 h fasting in *db/db* mice treated with vehicle or DC260126 for 3 weeks. And (C) HOMA-IR index was calculated in each group. Data are mean ± SE (n = 8 of each group). ** *p*<0.01, *** *p*<0.001 compared to lean group, # *p* <0.05 showed the significant differences between vehicle and DC260126-treated group.

**Figure 2:**
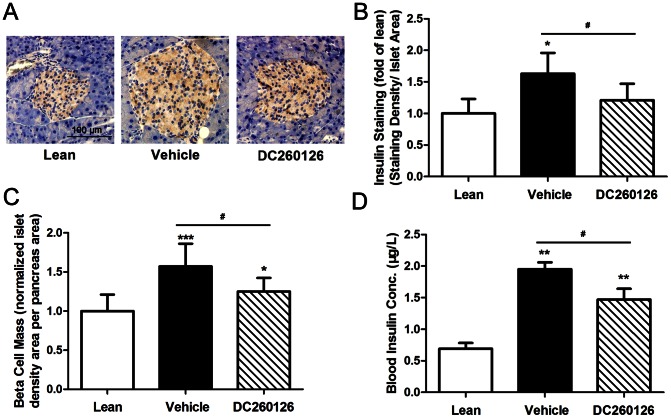
DC260126 reduces insulin expression in pancreatic β-cells and β-cell mass in *db/db* mice. (A) Representative immunostaining for insulin (marked with 3, 3′-diaminobenziding, brown) of 15 islets from eight independent individuals from each group. Scale bar  =  100 µm, and referred to all panels. (B) An obvious increased insulin staining in β-cells was observed in *db/db* vehicle mice (black bar) compared to lean littermates (white bar), however, 3 weeks treatment of DC260126 (slashed bar) significantly decreased the insulin expression in comparison with *db/db* vehicle mice. (C) DC260126 reduced the increasing of β-cell mass in *db/db* mice. (D) DC260126 significantly suppressed the elevated fast blood insulin concentration in *db/db* mice. Data are mean ± SE and * *p*<0.05, ** *p*<0.01, *** *p*<0.001 compared to lean group, # *p*<0.05 showed the significant differences between vehicle and DC260126-treated group.

After giving different dose of DC260126 to nine-week-old *db/db* mice for 5 days, the FSI level was decreased dose-dependently. And both 10 mg/kg and 30 mg/kg body weight could reduce FSI level of *db/db* mice significantly (Fig. 1A). For long term experiments, after giving 10 mg/kg body weight DC260126 to 6-week-old obese *db/db* male mice by tail vein injection once daily for 3 weeks, no significant alteration in overall body weight gain and daily food intake was found (Data not shown). And there was no obvious difference in levels of serum total cholesterol (TC) and triglyceride (TG) (Data not shown). The FPG level was also similar between DC260126 and vehicle group (Fig. 1B), indicating that 3 weeks administration of DC260126 in *db/db* mice could not exhibit any significant change in levels of blood glucose or lipids. However, the FSI level decreased significantly in DC260126-treated mice compared to vehicle-treated group (Fig. 2D). HOMA-IR index was calculated, and the HOMA-IR was found decreased in DC260126-treated mice compared to vehicle (Fig. 1C).

### DC260126 inhibits compensatory increase of insulin expression and β-cell mass

**Figure 3:**
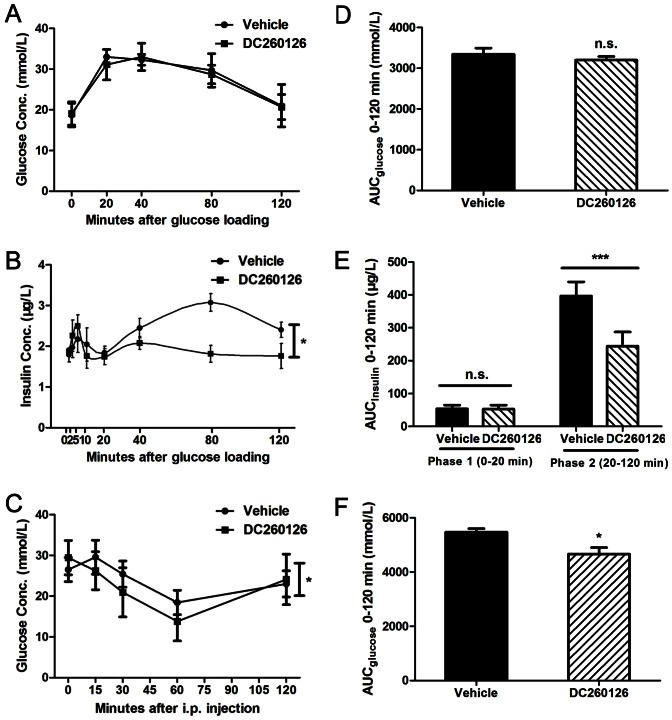
Effects of DC260126 on glucose tolerance, insulin secretion and insulin sensitivity in *db/db* mice. (A, D) No significant change was observed on blood glucose levels during the OGTT between vehicle and DC260126 treated mice. After intragastric administration with 2 g glucose per kg body weight at 0 min, blood glucose concentrations at the indicated times in the graphs. The area under curve (AUC) was calculated in right. (B, E) Serum insulin levels after intragastric administration of 2 g glucose per kg body weight. Insulin secretion during phase 1 (0 – 20 min) and phase 2 (20 – 120 min) was calculated by AUC. (C, F) Blood glucose levels at the indicated time following intraperitoneal injection of 1 U insulin per kg body weight in mice. AUC was shown in right. * indicated significant differences v.s. the vehicle group (n  =  8, * *p*<0.05, *** *p*<0.001).

During the pre-diabetic state, a compensatory phase is induced by peripheral insulin resistance in pancreatic β-cells. In this phase, β-cell mass and insulin output were increased [21]. Finally, constant increased secretion of insulin leads to β-cell exhaustion [21], [22]. Thus, preventing β-cells from overload might provide benefit for preventing β-cell failure. We found that DC260126 could lower fasted serum insulin levels (Fig. 2D) and alter glucose-stimulated insulin secretion (Fig. 3B) in *db/db* mice. To investigate the inhibitory effect of DC260126 on the compensatory increase of insulin expression and pancreatic β-cell mass, immunohistochemistry staining for insulin was performed. An increased insulin expression in the islets of Langerhans in *db/db* mice was found compared with their lean mates. In DC260126 treated *db/db* mice, however, insulin expression was decreased in islet compared with vehicle treated *db/db* mice (Fig. 2A and B). Moreover, a significant increase was found in pancreatic β-cell mass in 9-week-old *db/db* mice compared with their lean littermates, whereas the β-cell mass of DC260126 group was effectively lower than vehicle treated *db/db* mice after 3 weeks administration (Fig. 2C). These results provide strong evidence that DC260126 could prevent β-cells from overload to resist compensatory increasing in insulin expression, β-cell mass and serum insulin levels of *db/db* mice via inhibition of GPR40.

### Effect of DC260126 on blood glucose and insulin levels during OGTT and ITT in *db/db* mice

To further evaluate the effect of DC260126 on glucose metabolism and insulin secretion in *db/db* mice, OGTT and ITT were performed after 3 weeks administration of DC260126. Although the glucose levels during OGTT in DC260126-treated mice were similar to that in vehicle-treated mice (Fig. 3A and D), DC260126-treated mice showed an obvious decrease of blood glucose levels during ITT in response to insulin compared to the vehicle-treated group (Fig. 3C and F). It indicated that DC260126 could not alter glucose tolerance, but it improved insulin sensitivity in *db/db* mice after 3 weeks treatment. Meanwhile, the phase 2 of glucose-stimulated insulin secretion was obviously suppressed in DC260126-treated *db/db* mice in comparison with vehicle group (Fig. 3B and E). These data may provide evidence that DC260126 could reduce hyperinsulinemia and insulin resistance via inhibition of GPR40 in *db/db* mice.

### DC260126 reduces proinsulin/insulin ratio in *db/db* mice

**Figure 4:**
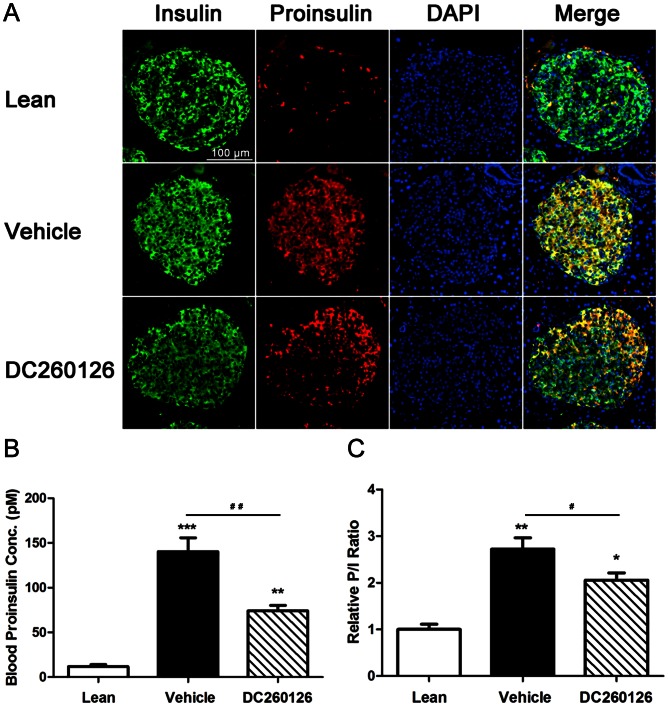
The expression of insulin/proinsulin and P/I ratio analysis in *db/db* mice. (A) Double immunofluorescence of insulin (green fluorescence) and proinsulin (red fluorescence) from pancreas in different groups, the merged figures were shown in right. The scale bar  =  100 µm, and referred to all panels. (B) The blood proinsulin concentrations were measured by ELISA kit, and proinsulin-to-insulin ratio were shown in the graph (C). All data are mean ± SE and * indicated significant differences (*p<0.05, ** *p*<0.01, *** *p*<0.001 compared to lean group, # *p*<0.05, ## *p*<0.01 showed the significant differences between vehicle and DC260126-treated group).

We had demonstrated that DC260126 could improve insulin sensitivity in Zucker fatty rats [16]. Similar effects were found in *db/db* mice (Fig. 2C and F). In long-term FFAs stimulation, hyperinsulinemia perturbs the activity of prohormone convertase 1/3 (PC1/3), one of the key enzymes that convert proinsulin to insulin [23]. Increased proinsulin-to-insulin (P/I) ratio is clarified to be one marker of β-cells dysfunction, has been observed in type 2 diabetes [24], [25], [26]. We examined the level of proinsulin in blood by ELISA, and immunohistochemistry was performed to observe the relative amount of proinsulin in pancreatic islets. As shown in Fig. 4A and B, proinsulin immunoreactivity in islets and the level of serum proinsulin were significantly increased in *db/db* mice compared to lean mice, and DC260126 treated group showed a lower value than vehicle treated *db/db* mice. Meanwhile, the P/I ratio of DC260126 treated mice is declined in both pancreatic islets and blood (Fig. 4A and C).

### DC260126 reduces pancreatic β-cells apoptosis in *db/db* mice

**Figure 5:**
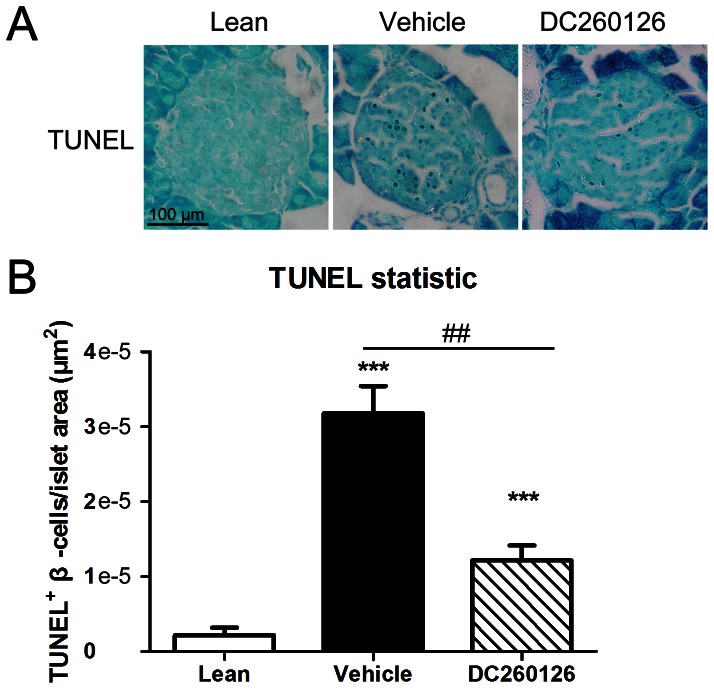
TUNEL staining showed that DC260126 reduced the number of apoptotic pancreatic β-cells. (A) The TUNEL positive cells were marked with dark brown in nucleis, and the sections were stained with methyl green, the scale bar  =  100 µm, and referred to all panels. (B) The number of TUNEL-positive cells was counted and divide with the area of each islet. At least 5 islets on each section from animals in the three groups were analyzed. ***p<0.001 v.s. lean group, and ## *p*<0.01 indicated significant differences between vehicle and DC260126-treated group.

In obesity-related diabetes, a sustained elevation of plasma FFAs leads to ER stress or oxidative stress in pancreatic β-cells, which induce β-cell apoptosis [27], [28]. The apoptotic rate of pancreatic β-cells in each group was compared. Fig. 5A showed an obvious increase in number of apoptotic β-cells in the islets of vehicle treated *db/db* mice, which indicated that with the development of hyperglycemia, the apoptotic pancreatic β-cells of *db/db* mice from age of 9-week-old increased significantly. However, treatment of DC260126 reduced the number of apoptotic β-cells significantly (Fig. 5B), which suggested that long-term administration of DC260126 could prevent pancreatic β-cells apoptosis.

## Discussion

Chronically elevated levels of FFAs lead to β-cell dysfunction, which is related to hyperinsulinemia, insulin resistance and glucose intolerance [29], [30]. As one of the FFA receptor, GPR40 is implicated in the pathophysiology of type 2 diabetes, and might become a new potential drug target for the treatment of the disease [8], [11], [31]. However, the role of GPR40 in the development of diabetes remains controversial, since several animal model studies showed inconsistent results [8], [10], [11].

Growing evidence indicated that some free fatty acids, such as palmitate could mobilize the Ca^2+^ release and amplify GSIS in β-cells via GPR40, but the chronic palmitate treatment could induce an increased risk of β-cell dysfunction [6], [32], [33]. Thus, a therapeutic approach based on GPR40 antagonism for the treatment of type 2 diabetes has been suggested [8], [34]. But other research showed opposite results that overexpression of GPR40 improved glucose tolerance in HFD diabetic mice [11]. And series of GPR40-selective small-molecule agonists have been developed for the purpose of type 2 diabetes medication in recent years [5], [12], [35]. The different transgenosis methods or diets used in the experiments might lead to these different observations of GPR40 agonism or antagonism [8], [10], [11].

However, the effects of GPR40 antagonists on type 2 diabetes were studied less. We had observed the effect of DC260126 on another hyperinsulinemia and insulin resistance model Zucker fatty rats [16]. There was no significant change on fasting blood glucose or glucose tolerance between control and DC260126 treat Zucker fatty rats, only the insulin sensitivity was improved and the hyperinsulinemia was reduced [16]. However, as we known, different from Zucker fatty rats, the diabetic phenomenon in *db/db* mice developed quickly, nine-week-old *db/db* mice could generate obviously hyperglycemia and insulin resistance [16], [36]. To investigate the effect of GPR40 antagonist DC260126 from pre-diabetes to diabetes in vivo, an obese diabetic *db/db* mice model was considered suitable in this study. Because the activation of GPR40 in intestine L cells could induce GLP-1 release and to enhance insulin secretion, the *db/db* mice in this experiment were given DC260126 via tail vein injection but not oral administration to reduce the possible effect of inhibition of GLP-1 release via antagonism of GPR40 in intestine. After 3 weeks administration of DC260126, no obvious change was observed neither in blood glucose nor lipids. We had reported that DC260126 could not improve glucose tolerance in Zucker fatty rats [16], combined with the results which Melkam et al. had proved in GPR40 knockout mice [37], the therapy based on GPR40 antagonism might not be effective enough to lower blood glucose levels. Nonetheless, these results are inconsistent with which Steneberg et al. had described [8]. We had also reported that DC260126 could protect MIN6 β-cells from chronic palmitate induced apoptosis via decreased ER stress [15]. Moreover, Abaraviciene et al. had also demonstrated that the adverse effects of palmitate on cultured islets could be counteracted by rosiglitazone, which could suppress GPR40-coupled signal transduction [34]. These studies suggested GPR40 antagonists should be useful in improving diabetic phenomenon. Thus, the unexpected results that DC260126 could not improve hyperglycemia may be explained by the pancreatic β-cells generate a compensatory phase during the prediabetic state in type 2 diabetes. During this phase, β-cell mass and insulin output increase to adapt elevated FFA levels in *db/db* mice [18], [38]. And the sustained hyperinsulinemia could induce β-cell dysfunction and related to insulin resistance [39]. GPR40 knockout mice could prevent HFD induced-hyperinsulinemia to keep a lower insulin level in the early stage of type 2 diabetes [8]. However, unlike the sustained period of GPR40 knockout mice, in this experiment, although the serum insulin levels were reduced, treatment of DC260126 might start too late or last too short to fail to reverse the process of hyperlipidemia and hyperglycemia in *db/db* mice. Meanwhile, the pharmacokinetics and the dose-response relationships of DC260126 might also contribute to this disappoint results. Besides, although we chose tail vein injection for DC260126 administration to minish the possible effect of DC260126 on GPR40 receptor in intestine, inhibition of GLP-1 release by DC260126 might also contribute to unchanged blood glucose level.

Interestingly, after 3 weeks administration of DC260126, an obvious improvement of insulin sensitivity was found in DC260126-treated *db/db* mice compared to vehicle-treated group. We had also found that DC260126 could reduce insulin resistance in Zucker fatty rats, and the phosphorylation level of AKT protein in liver was increased in DC260126-treated mice [6]. However, as known, GPR40 was less expressed in other tissues except pancreas and enteroendocrine cells [40]. Therefore, we speculated that DC260126 might have no direct effect on improving insulin sensitivity in peripheral tissues. Steneberg et al. had mentioned that the insulin sensitivity was improved in GPR40 knockout mice under HFD-treated condition [8]. Hyperinsulinemia is highly associated with insulin resistance in the development of T2D [39]. Recently study had also shown that limit peripheral hyperinsulinemia could provide benefit for the prevention of obesity and insulin resistance [41]. GPR40 is thought to contribute to obesity-induce hyperinsulinemia as it is required for FFA-stimulated insulin secretion. Thus, GPR40 knockout mice have a lower insulin level and would not develop hyperinsulinemia compared to normal mice on HFD-treatment [8]. In the current study, DC260126-treated mice got lower fasted serum insulin levels than vehicle-treated mice, and glucose-stimulated insulin secretion in DC260126-treated *db/db* mice were also decreased than vehicle group. It was in accord with the results that performed in GPR40 knockout mice in both Melkam et al. and Steneberg et al. reported [8], [37]. Therefore, like GPR40 knockout mice, DC260126 might reduce insulin tolerance mainly through decreasing hyperinsulinemia in *db/db* mice. Moreover, it has been proved that in the development of type 2 diabetes, hyperinsulinemia could induce an increasing proinsulin-to-insulin (P/I) ratio, and the increased level of proinsulin might be one of causes that induce insulin resistance in hyperinsulinemia [24], [25], [26]. We found that in DC260126-treated mice, there was a lower P/I ratio in comparison with vehicle-treated animals, demonstrating that the GPR40 antagonist improves insulin sensitivity at least partially through decreasing P/I ratio in *db/db* mice. Together, as both chronic hyperinsulinemia and increased P/I ratio could lead to insulin resistance [8], [24], the possible mechanism of improved insulin sensitivity by DC260126 might be related to reduced P/I ratio and integral prevention of compensatory development of insulin resistance caused by chronic hyperinsulinemia.

Meanwhile, a chronic effect of FFAs mediated β-cell lipotoxicity is mainly demonstrated by chronic fat accumulation in islets [4]. We speculated that inhibition of GPR40 by DC260126 could partially protect pancreatic β-cells from FFAs induced lipotoxicity and apoptosis. As we expected, treatment of DC260126 reduced the number of apoptotic β-cells in *db/db* mice, which might get a similar mechanism to the GPR40 antagonists did in MIN6 cells [15]. Reduced apoptosis of pancreatic β-cells might provide benefit for improving diabetic phenomenon, including insulin resistance. However, unlike FFAs, GPR40 agonists only activate GPR40 to amplify insulin secretion [12]. Thus, GPR40 agonists might lead to less lipotoxicity in pancreatic β-cells.

In conclusion, although the GPR40 antagonist, DC260126 might not be used for the treatment of type 2 diabetes, our data provided evidences that the theory under GPR40 antagonism for the treatment of type 2 diabetes might be reasonable in the early stage of type 2 diabetes.
